# Editorial: Safety and Effectiveness of COVID-19 Vaccines on COVID-19 Infection and Its Long-Term Consequences

**DOI:** 10.3390/vaccines13020120

**Published:** 2025-01-25

**Authors:** Ana Paula Junqueira-Kipnis

**Affiliations:** Institute of Public Health and Tropical Diseases, Federal University of Goiás, Goiânia CEP 74605-050, Brazil; ana_kipnis@ufg.br

COVID-19 continues to affect people around the world, according to the genomic reports. The SARS-CoV-2 Omicron variant continues to improve its ability to coexist with the human species, with Omicron XBB, JN.1, JN.1.7, KP.1, KP.1.18, KP.2, KP.3, KP.3.1.1, BA.2.86, and LB.1.2 being the variants of concern (VOCs) [1,2]. Most people infected with SARS-CoV-2 present mild to moderate symptoms and fever, and respiratory problems are the main concern. However, despite this, independent of health status, anyone can become sick and die. Additionally, long COVID-19 was observed in most COVID-19-affected persons with symptoms such as anosmia and dysgeusia, fatigue, shortness of breath, cognitive dysfunction, hair loss, chest pain, cough, myalgia, and respiratory disorders [3]. As of October 2024, more than 777 million cases were reported, and there were 7 million deaths. A total of 13.64 billion doses of COVID-19 vaccines were administered, culminating in a coverage of 67% of the world population, although only 37% received a booster dose. Still, almost 4 thousand deaths occurred monthly in 2024 [2].

The immune responses to COVID-19 vaccines among healthcare workers (HCWs) after the pandemic are important to understand the memory generated by the vaccines as well as by the disease that presents several lineages with different virulence that are being spread worldwide [1,2,4]. Revaccination of HCWs nowadays may help to strengthen the immune response against COVID-19 as the disease is still a respiratory problem, especially when associated with other comorbidities [5]. Thus, understanding the antibody and T lymphocyte-specific responses after the number of COVID-19 cases has diminished worldwide is going to help design new vaccines and address the implementation of revaccination schemes.

Healthcare workers (HCWs) remain the main group of individuals at risk of exposure to new COVID-19 VOCs. The immune response induced by the vaccines among HCWs was the focus of several studies; however, COVID-19 continues to cause an alarming number of cases, although the number of deaths reduced drastically after the implementation of specific vaccinations among HCWs. Thus, long-term immune response evaluation against COVID-19 and its incidence among HCWs will allow the understanding of the protection and the role of the vaccination during the pandemic and the disease burden. Strukcinskiene et al. evaluated the development of COVID-19 after vaccination (two doses, followed by a booster with Pfizer-BioNtech Comirnaty) in 534 HCWs. Most of the HCWs developed higher levels of antibodies against RBD after the vaccination that were reduced 6 months after the vaccination, but revaccination recovered the antibody levels up to 12 months. A total of 277 out of 534 HCWs developed COVID-19, and most of them were overweight or presented with obesity, although no correlation was observed with the antibody levels. Ultimately, 3 to 6 months after the last immunization, the majority of the HCWs became ill. Additionally, Matula et al. showed that HCWs vaccinated with BTN162b2 (Pfizer-BioNTech) and/or boosted with BBIBIP-CorV vaccines had COVID-19 (64.3%) up to one year after the last vaccination. Most of the participants presented specific antibody and T cell responses that were maintained one year after the vaccination or COVID-19 disease. Regardless of the levels or the maintenance of the immune response elicited against SARS-CoV-2, the majority of the HCWs did not present severe disease or have to be hospitalized. Strukcinskiene et al. and Matula showed that the immune response against COVID-19 was higher regardless of the type of booster received; however, none of the studies could differentiate if, after one year, the immune response observed was due to the vaccines or the exposure to COVID-19 infection, because the majority of the HCWs had COVID-19 about 6 months after the booster vaccination.

One robust study was conducted with 1220 HCWs in a tertiary hospital in Bucharest, Romania, by Chivu et al. Their work evaluated HCWs during 38 months of active surveillance and found that 767 HCWs had confirmed one COVID-19 episode and 221 HCWs had a second episode. Among the 767 COVID-19 cases, 20 were considered severe, and two people died of complications of chronic disease. However, regardless of the completion of vaccination, the COVID-19 incidence was similar, but the severity of the disease was associated with the absence of vaccination. The work conducted in Romania, where four different types of vaccines against COVID-19 were used (Comirnaty, Jcovden, Spikevax, and Vaxzevria), presented cases of severe COVID-19 (with hospitalization and deaths) that were not demonstrated by the other listed studies (Bucharest, Romania, and Budapest, Hungary). At this site, booster immunization had low adherence, but this fact was not associated with the severity of the disease. This result implies that although vaccines were a hallmark in reducing the number of deaths from COVID-19, the booster and the vaccine type might be important factors in reducing the disease burden even further. Additionally, vaccines that address VOCs need to be produced and implemented for at-risk populations with comorbidities associated with COVID-19 as well as be used in HCWs to reduce the disease burden.

BCG vaccination was shown to induce a long innate immune response that corroborates the protection against infection by the respiratory virus. In this sense, our group evaluated the efficacy of BCG to protect against COVID-19 among HCWs in a tertiary hospital [5]. Although no differences were observed in COVID-19 positivity between the non-vaccinated and vaccinated groups, it was observed that people with less symptomatic disease presented higher IFN-γ production, independently of BCG vaccination status, which prompted us to evaluate if the innate immune response of the vaccinated HCWs before vaccination was modifying the innate response induced by BCG. Indeed, da Costa et al. showed that HCWs who presented higher levels of NK cells producing IFN-γ before vaccination presented better responses to BCG, indicating that the previous immune status of the subject could influence the effectiveness of BCG vaccination.

Although in this Special Issue, long COVID-19 disease among HCWs was not evaluated, a case of an outbreak of COVID-19 after a festivity in Spain was evaluated in a clinical and laboratory setting after two or more vaccination doses for the development of long COVID-19. Domènech-Montoliu et al. observed that around 28% of COVID-19 patients had developed long COVID-19. Individuals that received the booster dose had a lower risk of developing COVID-19 complications due to the Omicron VOCs, confirming the importance of booster vaccination and the necessity of continuous vaccination, especially for new VOCs. Additionally, it was shown that COVID-19 vaccination followed by a booster with the BNT162b2 mRNA vaccine protected adolescents in a city in Japan, presenting an efficacy of 86.4%.

The COVID-19 pandemic had a significant impact on human lives; nonetheless, a large amount of data was gathered in the months following its outbreak that enabled an unprecedented rapid and efficient response. The studies involving HCWs definitely proved very useful, as they represented a cohort that was more susceptible to becoming infected. Vaccine studies with HCWs certainly helped to avoid many more deaths and COVID-19-related complications.
